# Farmers’ perceptions of bovine brucellosis in Benin

**DOI:** 10.14202/vetworld.2024.434-447

**Published:** 2024-02-23

**Authors:** Adeyemi Sharafa Dine Djibril, Fifa Théomaine Diane Bothon, Kadoeto Cyrille Boko, Bénoit Gbetondjingninougbo Koutinhouin, Souaibou Farougou

**Affiliations:** 1 Research Unit on Communicable Diseases, Polytechnic School of Abomey-Calavi, University of Abomey-Calavi, Cotonou, Benin; 2 Research Unit on Plant Extracts and Natural Flavors, Applied Chemistry Study and Research Laboratory, Polytechnic School of Abomey-Calavi, University of Abomey-Calavi, Cotonou, Benin; 3 Kaba Laboratory for Research in Chemistry and Applications; National Higher Institute of Industrial Technology, National University of Sciences, Engineering Technologies and Mathematics, Benin

**Keywords:** Benin, brucellosis risk factors, public health, knowledge

## Abstract

**Background and Aim:**

Cattle are the main source of meat in Benin. To improve the attitudes and practices of cattle breeders in relation to bovine brucellosis, a study has been carried out in Benin according to different agroecological zones. This study aimed to assess farmers’ knowledge and practices concerning bovine brucellosis to generate essential information for control programs and public health interventions.

**Materials and Methods:**

The study was conducted from February to May 2022, during which 608 farmers were interviewed using a structured questionnaire that provided information on socioeconomic characteristics, knowledge, and practices related to bovine brucellosis. Analysis of variance, Poisson regression, and the proportion comparison test were used to compare these characteristics in the different agroecological zones. At the end of the surveys, three distinct and homogeneous groups of perceptions (hierarchical classification of Multiple Correspondence Analysis components of R software) of bovine brucellosis were identified (these groups only consider farmers who declared knowledge of the disease). Groups were formed by applying the multiple correspondence analysis function of the FactoMineR library in R software, followed by a hierarchical ascending classification using the hierarchical clustering on principal component function of the same software (Agrocampus Rennes, France).

**Results:**

Only 38% of respondents were aware of brucellosis. Knowledge of brucellosis was not related to sex or education level but was higher among farmers in agroecological Zones 1 and 4. Ethnic Dendi herders (62.16%) had better knowledge of the disease than those from other sociolinguistic groups (Somba: 50%, Fulani: 40.91%, Baribas: 26.97%, and others: 8.82%). Reduced milk production (98.29%), presence of hygroma (87.18%), and abortion (56.84%) are the main signs reported by herders familiar with the disease. All three groups had good knowledge of the disease and its zoonotic nature. Groups 1 (96% of breeders) and 2 (2.14%) were aware of the risk factors (contact with affected animals, the consumption of raw milk, the handling of runts, and reproductive rejection). In the case of Brucella, they prefer to treat animals rather than sell them and use both traditional and modern medicines. Group 3 (1.71%) did not know the risk factors and preferred to sell animals in the event of illness.

**Conclusion:**

Pastoralists need to be made aware of the mode of transmission of bovine brucellosis, its clinical manifestations, its impact on animal health, and the zoonotic nature of the disease (impact on public health) so that bovine brucellosis can be rapidly detected in herds.

## Introduction

Brucellosis is a contagious bacterial disease that is endemic to many regions worldwide. It affects various animal species, including cattle [1, 2], and is caused by Gram-negative, non-spore-forming, non-encapsulated facultative intracellular coccobacillus belonging to the genus *Brucella* spp. [3, 4]. The *Brucella* genus comprises ten species that are classified according to host preferences and phenotypic differences [5, 6]. *Brucella abortus*, *Brucella melitensis*, and *Brucella suis* are the most important of these species [4].

Bovine brucellosis, which is mainly caused by *B. abortus*, causes major economic losses due to late pregnancy abortion, infertility, reduced livestock productivity (in this case, milk production), and slaughter of infected animals [5, 7–9]. In humans, brucellosis is clinically characterized by fever, excessive sweating, myalgia, and arthralgia (osteoarticular damage). Arthralgia is the most common complication of this disease. The course of the disease is often long-term and incapacitating [4, 10]. These pathogens have the potential to cause large-scale epidemics due to their low infectious dose, environmental resistance, and ability to spread through the air by aerosolization [4, 11]. Cattle and small ruminants excreting bacteria from milk and reproductive waste are the main sources of infection in humans and other animals [1, 11, 12].

Brucellosis is one of developing countries’ most serious animal diseases [12]. It is a major threat to human health, especially in low-income countries [10, 13–15]. However, it remains one of the most neglected zoonoses [16, 17]. Therefore, bovine brucellosis’s epidemiology must be understood to develop a disease control strategy.

Although brucellosis is common in developing countries [18], it is still under-reported and under-diagnosed. In West Africa, the incidence of bovine brucellosis significantly varies between countries. This variation has also been observed in herds of the same region [19, 20]. The prevalence of brucellosis in natural grazing systems is higher than that in urban and peri-urban systems [21, 22].

Akakpo *et al*. [23] conducted the first study of bovine brucellosis in Benin and reported a national seroprevalence of 10%. Several other cross-sectional studies were conducted between 2000 and 2005 and reported a brucellosis seroprevalence of up to 15.21% [24–26]. Noudeke *et al*. [27] observed an overall individual animal seroprevalence of 8.85%, with 19.33% for the Borgou regions (North of Benin) and 0% for the Atlantique department (South of Benin) in a study conducted in Benin’s main dairy basins from April to September 2015. Another study conducted by the same author on monthly variations in the prevalence of bovine brucellosis in Benin from February 2012 to January 2013 revealed a prevalence of up to 98.90% in the Gogounou commune [28]. The increase in the prevalence observed over time has led to interest in breeders’ perception of this disease and their proposal of improvements for integrated control.

It should be noted that none of these studies focused solely on the epidemiological aspects of the disease (determination of prevalence). In Benin, there has been no study on the socioeconomic aspects of bovine brucellosis (the perception of livestock farmers). Thus, the aim of this study was to assess the knowledge and perceptions of cattle breeders regarding the signs, mode of transmission, risk factors, prevention, and treatment of brucellosis. The findings of this study will contribute to the development of disease control strategies in Benin.

## Materials and Methods

### Ethical approval and Informed consent

The manuscript does not contain clinical studies or patient data, Ethical Committee approval was not required. All participants verbally consented before the survey began.

### Study period and location

**Figure-1:**
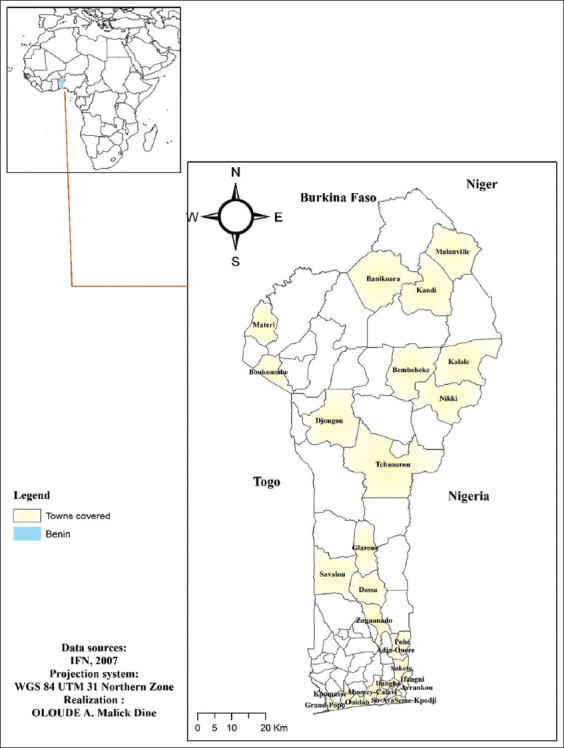
Study area. [Source: Data source: IFN 2007; Projection system: WGS 84 UTM Northern Zone; Realization: Oloude A. Malick Dine].

This study was conducted from February to May 2022 in Benin, West Africa. Benin covers an area of 114,763 km^2^ and is part of the intertropical zone. All Benin departments, except Couffo, were involved (Figure-1). It is located between 6°30’ and 12°30’ North and 1° and 3°40’ East longitude.

The country has three climatic zones: A Sudanian zone located between 9°45’ and 12°25’ N, a Sudan-Guinean zone located between 7°30’ and 9°45’ N, and a Guinean zone located between 6°25’ and 7°30’ N. The Sudanian zone has an average annual rainfall of <1000 mm, an average temperature of 27.5°C, a relative humidity of 54.9%, well-drained hydromorphic soils, and Savannah vegetation. On the other hand, in the Sudan-Guinean zone, rainfall is unimodal with an annual average of 900–1100 mm, average temperature varies between 21.2°C and 32.5°C, and relative humidity varies between 45.5% and 87.1%. The characteristic vegetation consists of a mosaic of open forest, dense forest, shrubby Savannah, and wooded Savannah with forest galleries. Finally, in the Guinean zone, rainfall is bimodal with an annual average of 1,200 mm, the average temperature varies between 25°C and 29°C, and the relative humidity is between 69% and 97%. The soils are either ferralitic or vertisols or rich in humus and minerals [29].

### Sample size

The study population consisted of cattle herds in Benin. The study sample size was 611 cattle herds.

We defined this sample size using Dagnelie’s formula [30]:


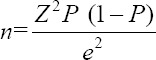


*n:* Herd/farm/breeder sample size;

*Z:* Confidence level set at 2.56 for (1 − α) = 99% with α (reliability threshold)of 1%;

*P:* Proportion of expected response set equal to 37.18 [28]

*e:* Acceptxtable margin of error set at 5%.

### Sampling method

This study is based on a three-stage survey. The first stage consisted of agroecological zones, the second stage consisted of communes, and the third stage consisted of the selection of cattle herds through breeders.

#### Selection of agroecological zones

Cattle herds have been sampled in the most important agroecological zones in terms of cattle breeding. Only agroecological zones with at least 15 herds were selected after a proportional distribution of herders across communes. Seven agroecological zones were selected for this purpose [31].

#### Selection of towns

On the basis of the total number of communes in each agroecological zone, a proportional distribution of the number of communes selected by agroecological zone was determined. The communes selected in each zone had the largest livestock herds. A total of 26 communes were initially identified before the sample size was allocated to the commune. However, only 24 samples were considered after removing agroecological zone 8, which did not have the required sample size.

#### Breeder selection

The herds were sampled from all 24 selected communes to ensure a wide range of information.

Cattle herds were distributed according to their size in each selected commune.

For each herd, the response units were cattle breeders. They were randomly selected from a list of cattle breeders held by veterinary officers in each of the study municipalities. A total of 608 breeders were selected.

### Data collection

Formal surveys using a structured questionnaire were used to collect data. Data on socioeconomic characteristics, farming system characteristics, knowledge of bovine brucellosis, and products and prices were collected. The questionnaire was digitized using the KoboToolBox (Kobo Inc, Massachusetts, United States) application and deployed on smartphones for the interviewers.

The questionnaire was administered through individual face-to-face interviews with each respondent. Fieldwork was conducted from February to May 2022.

### Analytical framework

#### Socioeconomic characteristics of respondents

The collected data enabled us to establish the sociodemographic characteristics of cattle breeders. Descriptive statistics were used to estimate parameters, such as mean and standard deviation for quantitative variables and absolute and relative frequency for qualitative variables. Analysis of variance (ANOVA), Poisson regression, and proportion comparison tests were used to compare continuous quantitative variables, count data, and qualitative variables in the different agroecological zones. The normality of the populations and homogeneity of their variances were checked beforehand, and the log transformation was applied to satisfy the application conditions. The Kruskal–Wallis test was used if it had no effect. Chi-square test of independence and Fisher’’s exact test were used to characterize breeders’ perceptions.

### Perception group analysis

Homogeneous perception groups on bovine brucellosis were constructed from qualitative perception variables using hierarchical classification of the components of multiple correspondence analysis (MCA). The optimal number of groups was determined using Calinski and Harabasz’s pseudo-F statistic. The most probable number of classes with the highest Calinski-Harabasz index value was the Jaccard bootstrap stability approach, which was then used to confirm the optimal number of perception groups for bovine brucellosis. In general, groups with a Jaccard stability value of <0.60 are considered unstable. It is considered very stable if its stability value is at least equal to 0.85.

Bovine brucellosis perception groups were then characterized using Fisher’s exact test for qualitative descriptors and ANOVA for continuous quantitative variables.

Quantitative variables counted were subjected to fish regression analysis.

### Statistical analysis

All collected data were entered and processed in a Microsoft Excel 2016 (Microsoft Corp., Washington, USA) spreadsheet. All statistical analyses were performed using R software version 4.0.4 (https://www.r-project.org) [32]. FactoMineR (http://factominer.free.fr) [33] and Factoextra packages (Marseille, France) (https://rpkgs.datanovia.com/factoextra/index.html) [34] were used for multivariate analysis.

## Results

### Socioeconomic characteristics of the respondents

#### Sociological characteristics of the respondents

Table-1 shows the socioeconomic characteristics of the respondents. The majority of respondents (98.19%) were male cattle farmers. Women comprised only 1.81% of the sample. These results are independent of the agroecological zone (p > 0.05). The majority of farmers surveyed had a low level of education. Three-quarters (75.16%) did not attend school, and only 6.91% completed primary education. The majority (90.79%) practiced Islam, and only 3.95% and 4.93 practiced Christianity and endogenous religions, respectively. The Fulani constituted the largest sociolinguistic group, with 72.37% of respondents. The rest of the respondents were Bariba (14.64%), Dendi (6.09%), Somba (1.32%), and others (5.59%). Most herders surveyed were married (46.38% monogamous and 41.12% polygamous, respectively).

**Table-1:** Socioeconomic characteristics of respondents by gender, level of education, religion, and ethnicity.

Variables	Agroecological zones							Total, N = 608	p-value

	Zone 1, N = 34 (5.6%)	Zone 2, N = 190 (31%)	Zone 3, N = 216 (36%)	Zone 4, N = 51 (8.4%)	Zone 5, N = 64 (11%)	Zone 6, N = 34 (5.6%)	Zone 7-8, N = 19 (3.1%)		
Gender									=0.9
Female	0 (0.00%)	6 (3.16%)	4 (1.85%)	0 (0.00%)	1 (1.56%)	0 (0.00%)	0 (0.00%)	11 (1.81%)	
Male	34 (100.00%)	184 (96.84%)	212 (98.15%)	51 (100.00%)	63 (98.44%)	34 (100.00%)	19 (100.00%)	597 (98.19%)	
Level of education									<0.001
Out of school	30 (88.24%)	137 (72.11%)	166 (76.85%)	47 (92.16%)	49 (76.56%)	18 (52.94%)	10 (52.63%)	457 (75.16%)	
Partial primary	4 (11.76%)	37 (19.47%)	35 (16.20%)	3 (5.88%)	9 (14.06%)	0 (0.00%)	0 (0.00%)	88 (14.47%)	
Completed primary	0 (0.00%)	14 (7.37%)	10 (4.63%)	0 (0.00%)	4 (6.25%)	10 (29.41%)	4 (21.05%)	42 (6.91%)	
Secondary 1^st^ cycle	0 (0.00%)	2 (1.05%)	4 (1.85%)	1 (1.96%)	2 (3.12%)	5 (14.71%)	2 (10.53%)	16 (2.63%)	
Secondary 2^nd^ cycle	0 (0.00%)	0 (0.00%)	1 (0.46%)	0 (0.00%)	0 (0.00%)	0 (0.00%)	1 (5.26%)	2 (0.33%)	
Superior	0 (0.00%)	0 (0.00%)	0 (0.00%)	0 (0.00%)	0 (0.00%)	1 (2.94%)	2 (10.53%)	3 (0.49%)	
Religion									<0.001
Christianity	1 (2.94%)	2 (1.05%)	11 (5.09%)	0 (0.00%)	1 (1.56%)	4 (11.76%)	5 (26.32%)	24 (3.95%)	
Islam	33 (97.06%)	179 (94.21%)	192 (88.89%)	50 (98.04%)	56 (87.50%)	30 (88.24%)	12 (63.16%)	552 (90.79%)	
Traditional	0 (0.00%)	9 (4.74%)	11 (5.09%)	1 (1.96%)	7 (10.94%)	0 (0.00%)	2 (10.53%)	30 (4.93%)	
Other	0 (0.00%)	0 (0.00%)	2 (0.93%)	0 (0.00%)	0 (0.00%)	0 (0.00%)	0 (0.00%)	2 (0.33%)	
Ethnic									<0.001
Bariba	0 (0.00%)	25 (13.16%)	46 (21.30%)	0 (0.00%)	18 (28.12%)	0 (0.00%)	0 (0.00%)	89 (14.64%)	
Dendi	3 (8.82%)	9 (4.74%)	20 (9.26%)	1 (1.96%)	3 (4.69%)	1 (2.94%)	0 (0.00%)	37 (6.09%)	
Fulani	29 (85.29%)	156 (82.11%)	143 (66.20%)	49 (96.08%)	35 (54.69%)	18 (52.94%)	10 (52.63%)	440 (72.37%)	
Somba	2 (5.88%)	0 (0.00%)	5 (2.31%)	1 (1.96%)	0 (0.00%)	0 (0.00%)	0 (0.00%)	8 (1.32%)	
Oyher	0 (0.00%)	0 (0.00%)	2 (0.93%)	0 (0.00%)	8 (12.50%)	15 (44.12%)	9 (47.37%)	34 (5.59%)	
Marital status									=0.041
Single with children	0 (0.00%)	8 (4.21%)	8 (3.70%)	0 (0.00%)	4 (6.25%)	1 (2.94%)	3 (15.79%)	24 (3.95%)	
Single without children	0 (0.00%)	8 (4.21%)	17 (7.87%)	0 (0.00%)	7 (10.94%)	3 (8.82%)	1 (5.26%)	36 (5.92%)	
Divorced	0 (0.00%)	0 (0.00%)	1 (0.46%)	0 (0.00%)	0 (0.00%)	0 (0.00%)	0 (0.00%)	1 (0.16%)	
Married monogamous	19 (55.88%)	93 (48.95%)	94 (43.52%)	23 (45.10%)	23 (35.94%)	18 (52.94%)	12 (63.16%)	282 (46.38%)	
Married polygamist	15 (44.12%)	78 (41.05%)	86 (39.81%)	28 (54.90%)	28 (43.75%)	12 (35.29%)	3 (15.79%)	250 (41.12%)	
Widower	0 (0.00%)	3 (1.58%)	10 (4.63%)	0 (0.00%)	2 (3.12%)	0 (0.00%)	0 (0.00%)	15 (2.47%)	

### Activities and years of experience of respondents

Table-2 shows the respondents’ different activities and their participation in the breeder groups. More than half of the respondents (50.38%) said that they belonged to a breeders’ group. Whether or not they belonged to a breeders’ association differed from one zone to another (p = 0.001), with 94.12% and 97.37% of breeders in Zone 1 and Zone 2, respectively. Agricultural activities: breeding (52.63%) and farming (35.20%) were the main activities carried out by respondents. These proportions differed significantly from one agroecological zone to another (p = 0.001). Other important activities listed include trade, handicrafts, public employment, private employment, and fish farming.

**Table-2:** Socioeconomic characteristics of respondents linked to membership of a breeder group and to the various activities carried out.

Variables	Agroecological zones							Total, N = 608	p-value

	Zone 1, N = 34 (5.6%)	Zone 2, N = 190 (31%)	Zone 3, N = 216 (36%)	Zone 4, N = 51 (8.4%)	Zone 5, N = 64 (11%)	Zone 6, N = 34 (5.6%)	Zone 7-8, N = 19 (3.1%)		
Group membership									<0.001
No	2 (5.88%)	5 (2.63%)	120 (55.56%)	30 (58.82%)	55 (85.94%)	25 (73.53%)	10 (52.63%)	247 (40.62%)	
Yes	32 (94.12%)	185 (97.37%)	96 (44.44%)	21 (41.18%)	9 (14.06%)	9 (26.47%)	9 (47.37%)	361 (59.38%)	
Main activity									<0.001
Civil servant	0 (0%)	0 (0%)	6 (2.78%)	0 (0%)	6 (9.37%)	2 (5.88%)	2 (10.53%)	16 (2.63%)	
Farmer	26 (76.47%)	61 (32.10%)	102 (47.22%)	10 (19.61%)	15 (23.44%)	0 (0%)	0 (0%)	214 (35.20%)	
Craftsman	0 (0%)	8 (4.21%)	8 (3.70%)	1 (1.96%)	1 (1.56%)	0 (0%)	1 (5.26%)	19 (3.13%)	
Retailer	0 (0%)	12 (6.32%)	7 (3.24%)	0 (0%)	0 (0%)	13 (38.24%)	4 (21.05%)	36 (5.92%)	
Breeder	7 (20.59%)	108 (56.84%)	93 (43.06%)	40 (78.43%)	42 (65.63%)	19 (55.88%)	11 (57.89%)	320 (52.63%)	
Private sector agent	1 (2.94%)	0 (0%)	0 (0%)	0 (0%)	0 (0%)	0 (0%)	1 (5.26%)	2 (0.33%)	
Fisherman	0 (0%)	1 (0.53%)	0 (0%)	0 (0%)	0 (0%)	0 (0%)	0 (0%)	1 (0.16%)	

Absolute frequency (relative frequency in %); mean (standard deviation)

Table-3 summarizes the adopted farming system, the average age of farmers, their experience in cattle farming, the size of their household and herd, and the number of assets. The vast majority (89.14%) of the herds sampled were sedentary. Almost one herder in 10 (9.54%) was transhuman, and only 1.32% were nomads. Sedentary herds were dominant in all agroecological zones except for agroecological zones 5 and 6, where transhumant herds were dominant. The average herd size was 33 individuals, with significantly larger (p = 0.001) herds in zones 7–8 and 4, with 63 and 43 heads of cattle, respectively.

**Table-3:** Socioeconomic characteristics of respondents linked to the breeding system.

Variables	Agroecological zones							Total, N = 608	p-value

	Zone 1, N = 34 (5.6%)	Zone 2, N = 190 (31%)	Zone 3, N = 216 (36%)	Zone 4, N = 51 (8.4%)	Zone 5, N = 64 (11%)	Zone 6, N = 34 (5.6%)	Zone 7-8, N = 19 (3.1%)		
Age (year)	37.79 (7.36)	42.61 (11.55)	43.73 (11.78)	43.88 (11.42)	47.16 (11.80)	38.41 (9.71)	41.05 (9.78)	43.04 (11.48)	<0.001
Experience (year)	19 (6.35)	24.59 (13.26)	21.97 (12.60)	29.47 (11.23)	22 (11.85)	12.74 (8.66)	9.89 (7.26)	22.36 (12.66)	<0.001
Household size	10.47 (4.22)	10.30 (6.09)	9.13 (5.14)	9.73 (4.21)	11.02 (6.90)	7.00 (3.58)	7.68 (5.98)	9.66 (5.57)	<0.001
Number of employees	4.41 (1.65)	3.63 (2.97)	3.48 (3.01)	3.90 (2.19)	4.06 (5.85)	4.50 (3.00)	5.37 (4.66)	3.79 (3.37)	<0.001
Herd size	30.24 (19.01)	34.81 (41.43)	26.95 (37.11)	42.45 (70.21)	31.75 (54.49)	35.24 (22.84)	62.89 (50.28)	32.78 (43.70)	<0.001

Absolute frequency (relative frequency in %); mean (standard deviation)

Their ages ranged from 18 to 77. The average age was 43 years, and the median age was 42 years. The oldest farmers (p = 0.001) were in agroecological zones 5 (48 years) and 3 and 4 (44 years), respectively. The youngest were in Zone 1, with an average age of 38.

They had an average of 22 years of experience in cattle breeding, with the greatest experience in agroecological zones 4, 2, 3, and 1. The size of their households ranged between 1 and 30 members, with an average of 10 members. The average household assets varied (p = 0.001) among the agroecological zones.

### Analysis of knowledge of bovine brucellosis

#### Knowledge of bovine brucellosis

The results of the analysis showed that only 38% of farmers were aware of bovine brucellosis. They also found that knowledge of bovine brucellosis was linked to the sociolinguistic group of the herder. The proportion of Dendi herders who were aware of bovine brucellosis (62.16%) was higher than that of Sombas (50%), Fulani (40.91%), Baribas (26.97%), and other sociolinguistic groups (8.82%). In addition, from a statistical point of view, as many men (38.69%) as women (27.27%) declared knowledge of bovine brucellosis, indicating that knowledge of bovine brucellosis was not gender related. In addition, this knowledge varies greatly according to the agroecological zones. In agroecological zones 1 and 4, more than half of the respondents indicated that they were aware of bovine brucellosis, whereas in zone 6, respondents were least aware. In addition, farmers who did not attend school were no less ignorant than those who did. Therefore, knowledge of bovine brucellosis is not related to schooling.

#### Knowledge of the signs of bovine brucellosis

Ninety-five percentage (95.73%) of farmers who knew about brucellosis recognized at least two signs, whereas only 46.58% knew about more.

### Awareness of abortion

Among farmers who were aware of bovine brucellosis, 56.84% recognized abortion as one of the signs of the disease. This perception is not related to gender or age. However, the sociolinguistic group, level of education, experience, membership of a herding group, and agroecological zone were influenced in different ways. Therefore, the proportion of herders who knew about abortion was higher among the Sombas and Fulani and lower among the Baribas, Dendis, and other sociolinguistic groups. Age-related knowledge about abortion appears to be a sign of bovine brucellosis. As age increased, so did the proportion of farmers who were aware of it. Finally, agroecological zones 2 and 3 were the areas where farmers were least aware of these signs.

#### Knowledge of the presence of hygromas

The presence of hygromas was perceived as a sign of bovine brucellosis by 87.18% of respondents who were aware of the disease. These perceptions did not vary according to sex, age, sociolinguistic group, education level, or herding group membership. However, it is related to experience in cattle breeding and agroecological zones. More than 70% of breeders were aware of this sign in all groups of experience. However, in agroecological zone 4, breeders knew the least about this sign.

#### Knowledge of the decline in milk production

A decline in milk production was the most common sign of bovine brucellosis among farmers. In fact, 98.29% of farmers surveyed were aware of this sign. These perceptions were not related to gender, age, sociolinguistic group, experience in cattle breeding, or membership of the breeders’ group. However, education level and agroecological zone were also influenced by this. Breeders with a lower level of education were more familiar with this sign, which was less familiar in agroecological zone 7.

### Knowledge of human risk factors

#### Knowledge of transmissibility

In general, 76.07% of farmers who were aware of bovine brucellosis were also aware of the possibility of its transmission to humans. Factors such as gender, age, level of education, and experience were not related to this perception, but sociolinguistic group, membership of a herding group, and agroecological zone were. The Fulani and Sombas were sociolinguistic groups with the greatest knowledge of this transmissibility. In addition, herders belonging to a herding group were more aware of the link between this factor and bovine brucellosis. In addition, the proportion of farmers with this knowledge was the highest in agroecological zones 1 and 4 but much lower in agroecological zone 7.

#### Contact and consumption of fresh milk

Almost all respondents who were aware of bovine brucellosis (98.29%) identified contact and consumption of fresh milk as risk factors for the disease. These perceptions were linked only to the level of education and the agroecological zones. These two perceptions were widely shared in all agroecological zones, with the exception of agroecological zone 7, where they were more limited.

#### Handling runts

Handling runts was identified as a risk factor by 54.27% of farmers who were aware of bovine brucellosis. This knowledge is related to age, education level, experience in cattle breeding, membership of breeders, and agroecological zone. As a result, the oldest, most experienced, and uneducated breeders had better knowledge of this risk factor than the youngest, least experienced, and educated breeders. On the other hand, breeders who do not belong to a breeders’ group seem to be more familiar with this factor. Finally, this perception was the least widespread in zones 2 and 7.

#### Meat consumption

Approximately 5% of farmers who were aware of bovine brucellosis indicated that the consumption of meat could be a factor in the transmission. This perception has only been linked to the agroecological zone, where it is only present in Zones 7 and 4.

### Behavior in the face of brucellosis

#### Treatment or sale of herd animals

All these breeders indicated that the entire herd should be systematically treated in the event of disease. However, 10.68% of respondents indicated that selling animals in the event of herd diseases would be preferable. This opinion is generally held by a few breeders belonging to a group of breeders.

### Prevention mode

Of all farmers who were aware of brucellosis, only 4.27% indicated that they did not take preventive measures. These farmers are mainly located in agroecological Zones 5 and 7. For the rest, the measures to be implemented should be aimed at avoiding disease within the herd, even if only 2% indicated that treatment should be envisaged.

Treatment of bovine brucellosis by breeders 89.74% of these farmers reported using plants to treat bovine brucellosis. This practice is more common among farmers and less common in the last two agroecological zones. Almost all respondents (95.30%) treated bovine brucellosis with veterinary medicine, whereas farmers in agroecological Zone 7 did not use it.

### Group analysis of perceptions of bovine brucellosis in Benin

**Figure-2:**
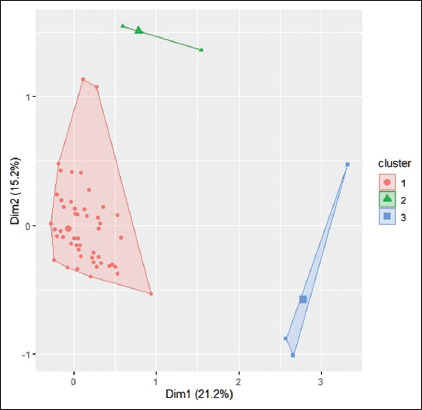
Typology of cattle breeders in Benin based on brucellosis-related knowledge.

The MCA results indicated that the first two constructed dimensions explained only 36.44% (Figure-2) of the total variance, whereas seven explained at least 80%.

As a result of the hierarchical classification of the components of the ACM, three distinct and homogeneous perceptions of bovine brucellosis were identified.

Group 1 consists of breeders who know the signs (abortion, decrease in milk production, and presence of hygromas), risk factors for humans (contact, handling of runts, and consumption of raw milk) (Table-4), who are unwilling to sell animals in the event of illness, prefer to care for them, and who believe that disease control is the best way to preserve the herd and use veterinary care and plants to treat the disease (Table-5). This perception group is the largest, accounting for 96.15% of farmers. Therefore, it reflects the most common perception of bovine brucellosis in Benin. The breeders in this group had an average age of 45, an average experience of 25 years, and an average herd size of 44 animals. Most breeders (96%) were sedentary. All women and most men (96.10%) belonged to this category. In terms of sociolinguistic groups, group 1 comprises some Baribas, Dendis, and Sombas, 76.44% Fulani, and 0.89% other sociolinguistic groups. In addition, it includes 76% of unschooled herders and 24.00% of schooled herders (Table-6).

**Table-4:** Knowledge of clinical signs of bovine brucellosis.

Characteristics	Group 1, N = 225 (96.15%)	Group 2, N = 5 (2.14%)	Group 3, N = 4 (1.71%)	Total, N = 234	p-value
Knowledge of transmissibility					=0.004
No	51 (22.67%)	1 (20.00%)	4 (100.00%)	56 (23.93%)	
Yes	174 (77.33%)	4 (80.00%)	0 (0.00%)	178 (76.07%)	
Contact					<0.001
No	20 (8.89%)	0 (0.00%)	4 (100.00%)	24 (10.26%)	
Yes	205 (91.11%)	5 (100.00%)	0 (0.00%)	210 (89.74%)	
Raw milk consumption					<0.001
No	0 (0.00%)	0 (0.00%)	4 (100.00%)	4 (1.71%)	
Yes	225 (100.00%)	5 (100.00%)	0 (0.00%)	230 (98.29%)	
Handling runts					=0.005
No	103 (45.78%)	0 (0.00%)	4 (100.00%)	107 (45.73%)	
Yes	122 (54.22%)	5 (100.00%)	0 (0.00%)	127 (54.27%)	
Meat consumption					<0.001
No	218 (96.89%)	5 (100.00%)	0 (0.00%)	223 (95.30%)	
Yes	7 (3.11%)	0 (0.00%)	4 (100.00%)	11 (4.70%)	

**Table-5:** Brucellosis behavior.

Characteristics	Group 1, N = 225 (96.15%)	Group 2, N = 5 (2.14%)	Group 3, N = 4 (1.71%)	Total, N = 234	p-value
Sale of affected animals					>0.9
No	200 (88.89%)	5 (100.00%)	4 (100.00%)	209 (89.32%)	
Yes	25 (11.11%)	0 (0.00%)	0 (0.00%)	25 (10.68%)	
Treatment in case of illness					
Yes	225 (100.00%)	5 (100.00%)	4 (100.00%)	234 (100.00%)	
No preventive action					<0.001
No	221 (98.22%)	0 (0.00%)	3 (75.00%)	224 (95.73%)	
Yes	4 (1.78%)	5 (100.00%)	1 (25.00%)	10 (4.27%)	
Preventive action					<0.001
No	0 (0.00%)	5 (100.00%)	1 (25.00%)	6 (2.56%)	
Yes	225 (100.00%)	0 (0.00%)	3 (75.00%)	228 (97.44%)	
Phytotherapy					=0.002
No	20 (8.89%)	1 (20.00%)	3 (75.00%)	24 (10.26%)	
Yes	205 (91.11%)	4 (80.00%)	1 (25.00%)	210 (89.74%)	
Veterinary care					=0.001
No	6 (2.67%)	1 (20.00%)	4 (100.00%)	11 (4.70%)	
Yes	219 (97.33%)	4 (80.00%)	0 (0.00%)	223 (95.30%)	

Absolute frequency (relative frequency in %); mean (standard deviation)

**Table-6:** Bovine brucellosis perception groups in Benin.

Characteristics	Group 1 N = 225 (96.15%)	Group 2 N = 5 (2.14)	Group 3 N = 4 (1.71)	Total N = 234	p-value
Gender					>0.9
Female	3 (1.33)	0 (0.00)	0 (0.00)	3 (1.28)	
Male	222 (98.67)	5 (100.00)	4 (100.00)	231 (98.72)	
Ethnic group					=0.3
Other	2 (0.89)	0 (0.00)	1 (25.00)	3 (1.28)	
Bariba	24 (10.67)	0 (0.00)	0 (0.00)	24 (10.26)	
Dendi	23 (10.22)	0 (0.00)	0 (0.00)	23 (9.83)	
Fulani	172 (76.44)	5 (100.00)	3 (75.00)	180 (76.92)	
Somba	4 (1.78)	0 (0.00)	0 (0.00)	4 (1.71)	
Level of education					=0.017
Out of school	171 (76.00)	4 (80.00)	3 (75.00)	178 (76.07)	
Complete primary	7 (3.11)	0 (0.00)	0 (0.00)	7 (2.99)	
Partial primary	45 (20.00)	0 (0.00)	0 (0.00)	45 (19.23)	
Secondary 2^nd^ cycle	0 (0.00)	0 (0.00)	1 (25.00)	1 (0.43)	
Secondary 1^st^ cycle	2 (0.89)	1 (20.00)	0 (0.00)	3 (1.28)	
Group membership					=0.7
No	53 (23.56)	0 (0.00)	1 (25.00)	54 (23.08)	
Yes	172 (76.44)	5 (100.00)	3 (75.00)	180 (76.92)	
Agro-ecological zone					<0.001
Zone 1	20 (8.89)	0 (0.00)	0 (0.00)	20 (8.55)	
Zone 2	71 (31.56)	0 (0.00)	0 (0.00)	71 (30.34)	
Zone 3	90 (40.00)	0 (0.00)	0 (0.00)	90 (38.46)	
Zone 4	27 (12.00)	0 (0.00)	0 (0.00)	27 (11.54)	
Zone 5	13 (5.78)	4 (80.00)	0 (0.00)	17 (7.26)	
Zone 6	3 (1.33)	0 (0.00)	0 (0.00)	3 (1.28)	
Zone 7	1 (0.44)	1 (20.00)	4 (100.00)	6 (2.56)	
Age group					=0.2
18–40 years old	76 (33.78)	1 (20.00)	1 (25.00)	78 (33.33)	
40–62 years	129 (57.33)	2 (40.00)	3 (75.00)	134 (57.26)	
Over 62	20 (8.89)	2 (40.00)	0 (0.00)	22 (9.40)	
Level of experience					=0.062
<15	44 (19.56)	1 (20.00)	2 (50.00)	47 (20.09)	
15–30 years	95 (42.22)	0 (0.00)	2 (50.00)	97 (41.45)	
30–45 years	65 (28.89)	2 (40.00)	0 (0.00)	67 (28.63)	
Over 45	21 (9.33)	2 (40.00)	0 (0.00)	23 (9.83)	
Age (year)	45.61 (11.21)	54.20 (18.10)	48.50 (12.26)	45.84 (11.40)	=0.3
Experience (year)	25.27 (12.80)	35.00 (21.21)	15.25 (8.77)	25.30 (13.04)	=0.13
Herd size (number)	44.21 (55.70)	190.00 (74.16)	92.50 (74.22)	48.15 (60.24)	<0.001

Group 2 is made up of farmers who are aware of the signs (abortion, drop in milk production, and presence of hygromas), the risk factors for humans (contact, handling of runts, and consumption of raw milk), who are unwilling to sell their animals in the event of illness and prefer to treat them, and who do nothing to prevent the disease but have recourse to both veterinary care and the use of plants to treat the disease (Tables-4 and 5). This group represents only 2.14% of breeders, reflecting a rather rare perception. The breeders in this group had an average age of 54 years, an average experience of 35 years, and an average herd size of 190 animals (Table-6).

Women do not belong to this group, and only men are represented. This group consists of only Fulani. In addition, 80.00% of unschooled herders and 20.00% of schooled herders are classified (Table-6).

Group 3 is made up of breeders who are aware of the signs (abortion, drop in milk production, and presence of hygromas), who are unaware of the risk factors for humans (contact, handling of runts, and consumption of raw milk), who are unwilling to sell their animals in the event of illness, preferring to look after them; who believe that disease control is the best way of preserving the herd, and do not resort to veterinary care or the use of plants to treat disease. This group consisted of only 1.71% of breeders. The breeders in this group have an average age of 48 years, 15 years of experience and an average herd size of 92 animals and are engaged in sedentary and transhuman farming. Women do not belong to this group, and only men are represented (Table-6). This group comprises only 75.00% Fulani and 25.00% other sociolinguistic groups. In addition, 75% of unschooled herders and 25.00% of schooled herders are educated (Table-6).

## Discussion

### Socioeconomic characteristics of respondents

This study demonstrates that livestock farming is essentially a male activity. This finding was confirmed by Djohy *et al*. [35] and Hessa *et al*. [36] in Benin, as well as in several African countries such as Kenya [37], Tanzania [38], and Cameroon [39], with rates between 69% and 92% of men engaged in cattle breeding. The Fulani comprised the largest sociolinguistic group. This confirms that Fulani social organization attributes cattle breeding to men [40, 41]. Farming is the second most important occupation of respondents. Dahouda *et al*. [42] reported an association between agriculture and livestock farming in Benin’s cattle-raising systems, which increases their resilience to external changes (herd diseases and drought). Houndje *et al*. [43] confirmed the low educational level of the farmers surveyed in several geographical areas of Benin Hessa *et al*. [36] also found a very low literacy/education rate similar (15%) to ours during their studies in the cotton-growing areas of Benin. The majority of herders (90.79%) practiced Islam. The majority adherence to Islam was observed by Youssef *et al*. [44]. The average age of the breeders surveyed was 43 years old, with an average of 22 years of experience in cattle breeding. In addition, most respondents were sedentary and owned an average of 33 cattle. This herd size is lower than that reported by Chabi Toko [45] in cattle farms in northeastern Benin and Djohy *et al*. [35] in central Benin. Differences in age, years of experience, and type of farming (transhumant versus sedentary in our study) may account for these differences. Contrary to Djohy *et al*. [35], more than half of the respondents belonged to cattle breeders’ associations.

### Analysis of knowledge of bovine brucellosis

Assessing the level of herders’ knowledge of the disease is essential for developing and implementing more effective awareness programs and brucellosis control initiatives to meet the needs and perspectives of local communities [46]. To the best of our knowledge, this is the first study to examine herders’ level of knowledge about bovine brucellosis in Benin. The data collected show that only a third of the herders interviewed were aware of bovine brucellosis. Kang’ethe *et al*. [47] reported a similar level of knowledge (30%) in Kenya. Furthermore, Kansiime *et al*. [48] in south-western Uganda, Edao *et al*. [49] in Ethiopia, Cloete *et al*. [50] in South Africa, Cadmus *et al*. [51] in Nigeria, Madzingira *et al*. [52] in Namibia, and Babo *et al*. [53] in Côte d’Ivoire also reported low awareness of brucellosis among livestock farmers. Buhari *et al*. [54] reported high levels (93%) of awareness of brucellosis among cattle farmers in Kaduna State, northern Nigeria. Nabirye *et al*. [55] in northern Uganda, Obonyo and Gufu [56] in Kenya, and Musallam *et al*. [57] in Jordan also estimated that 63%, 79%, and 100% of respondents (mostly farmers) had heard of brucellosis, respectively. The fact that you know about the disease is not synonymous with knowledge of it. This is due to the lack of knowledge of the transmissibility of the disease, its zoonotic nature, and the associated risk factors reported by the same authors. These differences in knowledge in Africa and in other parts of the world reflect a significant variability in the distribution of information and the functioning of veterinary services in different regions.

The low level of knowledge recorded during the present study could be linked to the low level of contact between farmers and the germ responsible for brucellosis (*Brucella* spp.). The relatively low prevalence rates (between 0% and 19.33%) [24–26, 28] recorded in Benin since the 2000s could explain the low level of awareness about the disease among breeders. Dez and Coelho [58] confirmed these hypotheses by reporting a positive correlation between experience of brucellosis in cattle, disease prevalence, and knowledge of brucellosis among herders.

The level of education had little influence on the knowledge of the disease. This finding is contrary to the observations of several authors in Africa (Mufinda *et al*. [59] in Angola, Njuguna *et al*. [60] in Kenya), Asia (Lindahl *et al*. [61] in Tajikistan; Arif *et al*. [62] in Pakistan, and Kothalawala *et al*. [63] in Sri Lanka), and Latin America [64], who have reported a positive correlation between knowledge of the disease and level of education.

Farmers in agroecological zones 1 and 4 have the best knowledge of the disease. These agroecological zones largely correspond to the departments of Alibori and Atacora, two of which have the largest cattle populations [65]. The low level of knowledge of cattle breeders surveyed could jeopardize public health because of erroneous practices in handling runts and reproductive waste and in handling and cooking meat. According to Kunda *et al*. [66], a lack of knowledge about brucellosis is an obstacle to the control and elimination of the disease. Raising awareness of brucellosis and brucellosis-related knowledge among professionals is an important aspect of effective brucellosis control [67]. Therefore, there is an urgent need to raise awareness of bovine brucellosis among farmers and all stakeholders in the bovine value chain.

The main signs of bovine brucellosis are abortion, reduced milk production, and hygroma. [53, 56, 60, 68]. In this study, reduced milk production was the breeders’ most recognized clinical sign. Alsaif *et al*. [69] and Ducrotoy *et al*. [70] have reported that reduced milk production is one of the main consequences of *Brucella* infection in cattle. In contrast to the findings of our study, farmers reported abortion as the main sign of brucellosis in Nigeria [38, 54, 71]. In view of the different levels of knowledge of the disease, this slight difference between farmers from different countries or regions is not surprising. The low level of knowledge of brucellosis among many breeders and the transfer of information about the disease from generation to generation or from breeder to breeder may contribute to the spread of brucellosis to the detriment of brucellosis to the detriment of brucellosis to the detriment of brucellosis [53]. For example, Kiros *et al*. [72] reported a wide variation in mortality rates (between 30% and 80%) in susceptible herds.

Previous experience in cattle breeding has a significant impact on farmers’ awareness of abortion as a clinical sign of brucellosis. The fact that the recognition of this sign evolves positively with age suggests that older farmers have experienced brucellosis.

In addition to reduced milk production, hygroma (87.18%) and abortion (56.84%) were the other signs mentioned by my respondents. Ntirandekura *et al*. [38] mentioned some additional signs, such as fever, hygroma, vaginal discharge, lack of appetite, orchitis, fatigue, and general weakness.

### Knowledge of human risk factors and brucellosis behavior

During our investigations, almost all respondents recognized contact and consumption of unpasteurized milk as risk factors. In addition, three-quarters of farmers and one in two farmers have recognized that bovine brucellosis is a zoonotic disease and that unprotected handling of runts and fetal material is also a risk factor. Cloete *et al*. [50] confirmed that consumption of unpasteurized milk (66.7%), assisting in calving or handling the unprotected placenta (22.2%), slaughtering an infected animal (16.7%), and handling the runt (11.1%) are the greatest risk factors for hepatitis. According to Kansiime *et al*. [48] and Tialla *et al*. [73], the consumption of unpasteurized raw milk or its products (unpasteurized curdled milk, cheese), assistance with abortions, and unprotected handling of runts greatly increases the risk of human exposure. In the case of *Brucella* infection, the udder is significantly affected, which facilitates the excretion of the bacterium into the milk [74, 75]. In addition, 5% of farmers believe that the consumption of brucellosis-infected animal meat is also a source of contamination. According to Ntirandekura *et al*. [38], the consumption of raw meat, unpasteurized milk, and unprotected assistance of animals during parturition are risk factors for the transmission of bovine brucellosis to humans. Apart from these risk factors, Adesokan *et al*. [76] also mention cohabitation with animals and lack of hygiene as significant risk practices in the transfer of *Brucella* infection from animals to humans.

The risk of transmission associated with handling runts is better known among breeders who are not members of the breeding group. This result reflects the lack of general information available to all breeders and the poor circulation of information within these groups. It could also indicate the circulation of contradictory information, which prevents breeders from obtaining clear and accurate information.

This study shows that in the event of brucellosis, farmers resort to treating the entire herd. Therefore, 90% indicated that bovine brucellosis was treated using plants and veterinary products. One in ten respondents said that in case of herd disease, it would be preferable to sell the animals. Kansiime *et al*. [48] reported that 84% of farmers use veterinary care for the treatment of animal pathologies. Cloete *et al*. [50] added that almost half of the farmers surveyed were referred to veterinary services for assistance. As shown in this study, there is no link between education level and knowledge of disease. Madzingira *et al*. [52] and Mangesho *et al*. [77] pointed out that veterinary agents and other animal health actors who were aware of the disease do not have different practices from farmers who were unaware of the disease. This suggests that all stakeholders in the cattle value chain should be fully aware of the risks associated with this disease and the best practices to be adopted.

## Conclusion

Knowledge of diseases is a key step toward the development of prevention and control measures. The results show that farmers have little knowledge about bovine brucellosis and its risk factors. In view of the economic impact of this disease on herd reproductive health and milk production, several recommendations should be made to minimize the risk of spreading this disease. These include raising awareness among farmers, livestock traders, and processors of good animal production and processing practices. In conclusion, further studies are required to determine the impact of endogenous practices on reducing the spread of the disease.

## Authors’ Contributions

ASDD, FTDB, and KCB: Conceived the study design and collected the data. ASDD, SF, and BGK: Analyzed the data and wrote the manuscript. BGK and SF: Corrected the manuscript. All authors have read, reviewed, and approved the final manuscript.

## References

[1] Nejad R.B, Krecek R.C, Khalaf O.H, Hailat N, Arenas-Gamboa A.M (2020). Brucellosis in the Middle East:Current situation and a pathway forward. PLoS Negl. Trop. Dis.

[2] Getahun T, Urge B, Mamo G (2023). Seroprevalence of bovine brucellosis in selected sites of central highland of Ethiopia. Vet. Med. Res. Rep.

[3] Gwida M, Al Dahouk S, Melzer F, Rösler U, Neubauer H, Tomaso H (2010). Brucellosis-regionally emerging zoonotic disease?. Croat. Med. J.

[4] Seleem M.N, Boyle S.M, Sriranganathan N (2010). Brucellosis:A re-emerging zoonosis. Vet. Microbiol.

[5] Nicoletti P (2010). Brucellosis:Past, present and future. Prilozi.

[6] Godfroid J, Cloeckaert A, Liautard J.P, Kohler S, Fretin D, Walravens K, Garin-Bastuji B, Letesson J.J (2005). From the discovery of the Malta fever's agent to the discovery of a marine mammal reservoir, brucellosis has continuously been a re-emerging zoonosis. Vet. Res.

[7] Boukary A.R (2013). Epidemiology of animal brucellosis and tuberculosis in the urban, suburban and rural areas in Niger [Epidémiologie De La Brucellose Et De La Tuberculose Animales Dans Les Milieux Urbain, Périurbain Et Rural Au Niger] (Ph.D. in Veterinary Sciences). University of Liège (ULiège).

[8] El-Diasty M, El-Said R, Abdelkhalek A (2021). Seroprevalence and molecular diagnosis of sheep brucellosis in Dakahlia governorate, Egypt. Ger. J. Vet. Res.

[9] Ferreira B.F.S, Barros M.L, Ferreira F, Rocha A, Dias R.A, Filho J.H.H.G, Heinemann M.B, Telles E.O, Alevate G.C, Neto J.S.F (2023). Economic analysis of bovine brucellosis control in the Rondônia state, Brazil. Trop. Anim. Health Prod.

[10] Jin M, Fan Z, Gao R, Li X, Gao Z, Wang Z (2023). Research progress on complications of Brucellosis. Front. Cell. Infect. Microbiol.

[11] Tazerart F, Aliouane K, Grine G (2022). Evolution of animal and human brucellosis in Algeria:A mini-narrative review. New Microbes New Infect.

[12] Selim A, Gaber A, Moustafa A (2015). Diagnosis of brucellosis in ruminants in Kafr El-Sheikh governorate, Egypt. Int. J.

[13] Kefaloudi C, Mellou K, Dougas G, Vorou R, Mitrou K, Kontopidou F (2022). Human brucellosis in Greece, 2005–2020:A persistent public health problem. Vector Borne Zoonotic Dis.

[14] Burns R.J.L, Le K.K, Siengsanun-Lamont J, Blacksell S.D (2023). A review of coxiellosis (Q fever) and brucellosis in goats and humans:Implications for disease control in smallholder farming systems in Southeast Asia. One Health.

[15] Dahourou L.D, Ouoba L.B, Minoungou L.B.G, Tapsoba A.R.S, Savadogo M, Yougbaré B, Traoré A, Bada Alambédji R (2023). Prevalence and factors associated with brucellosis and tuberculosis in cattle from extensive husbandry systems in Sahel and Hauts-Bassins regions, Burkina Faso. Sci. Afr.

[16] Jiang H, O'Callaghan D, Ding J.B (2020). Brucellosis in China:History, progress and challenge. Infect. Dis. Poverty.

[17] Khezzani B, Narimane Aouachria A, Khechekhouche E.A, Djaballah S, Djedidi T, Bosilkovski M (2021). Epidemiological characteristics of human brucellosis in the province of El-oued (south-eastern Algeria) [Caractéristiques épidémiologiques de la brucellose humaine dans la province d'El-Oued, sud-est algérien]. SantéPublique.

[18] Holt H.R, Bedi J.S, Kaur P, Mangtani P, Sharma N.S, Gill J.P.S, Singh Y, Kumar R, Kaur M, McGiven J (2021). Epidemiology of brucellosis in cattle and dairy farmers of rural Ludhiana, Punjab. PLoS Negl. Trop. Dis.

[19] Akakpo J.A, Ndour A.P.N (2013). Bovine brucellosis in West and Central Africa:a review [La brucellose bovine en Afrique de l'ouest et du centre:État des lieux]. Rev. Afr. San. Prod. Anim [Rev. D'élevage Méd. Vét. Pays Trop.].

[20] Cadmus S.I, Alabi P.I, Adesokan H.K, Dale E.J, Stack J.A (2013). Serological investigation of bovine brucellosis in three cattle production systems in Yewa Division, south-western Nigeria. J. S. Afr. Vet. Assoc.

[21] Makita K, Fèvre E.M, Waiswa C, Eisler M.C, Thrusfield M, Welburn S.C (2011). Herd prevalence of bovine brucellosis and analysis of risk factors in cattle in urban and peri-urban areas of the Kampala economic zone, Uganda. BMC Vet. Res.

[22] Megersa B, Biffa D, Abunna F, Regassa A, Godfroid J, Skjerve E (2011). Seroprevalence of brucellosis and its contribution to abortion in cattle, camel, and goat kept under pastoral management in Borana, Ethiopia. Trop. Anim. Health Prod.

[23] Akakpo A.J, Bornarel P, D'Almeida J.F (1984). Cattle brucellosis epidemiology in tropical Africa. 1. Serological survey in the People's Republic of Benin [Epidémiologie de la brucellose bovine en Afrique tropicale. I. Enquête sérologique en République Populaire du Bénin]. Rev. Elev. Med. Vet. Pays Trop. [Rev. D'élevage Méd. Vét. Pays Trop.].

[24] Koutinhouin B, Youssao A.K.I, Houehou A.E, Agbadje P.M (2003). Prevalence of bovine brucellosis in the traditional breedings supported by the PDE (Projet pour le Développement de l'Elevage) in Benin [Prevalence de la brucellose bovine dans les elevages traditionnels encadres par le Projet pour le Developpement de l'Elevage (PDE) au Benin]. Rev. Méd. Vét.

[25] Adehan R, Koutinhouin B, Baba-Moussa L.S, Aigbe L, Agbadje P.M, Youssao A.K.I (2005). Prevalence of bovine brucellosis in Benin states farm from 2000 to 2003 [Prévalence de la brucellose bovine dans les fermes d'Etat du Bénin de 2000 à2003]. Rev. Afr. San. Prod. Anim.

[26] Vikou R, Aplogan L.G, Ahanhanzo C, Baba-Moussa L, Gbangboche A.B (2018). Prevalence of bovine brucellosis and tuberculosis in Benin [Prévalence de la brucellose et de la tuberculose chez les bovins au Bénin]. Int. J. Biol. Chem. Sci.

[27] Noudeke N.D, Dossou-Gbete G, Pomalegni C, Mensah S, Aplogan L.G, Atchadé G, Dougnon J, Youssao I, Mensah G.A, Souaïbou F (2017b). Prevalence of bovine brucellosis, tuberculosis and dermatophilosis among cattle from Benin's main dairy basins. J. Vet. Med. Anim. Health.

[28] Noudeke N.D, Aplogan L.G, Dossa F, Youssao I, Farougou S (2017a). Monthly variations of the prevalence of bovine brucellosis in Benin. Adv. Anim. Vet. Sci.

[29] Gnanglè C.P, Kakaï R.G, Assogbadjo A.E, Vodounnon S, Yabi J.A, Sokpon N (2011). Past climate trends:modeling, local perceptions and adaptation in Benin [Tendances climatiques asses, modélisation, perceptions et adaptations locales au Bénin]. Climatologie.

[30] Dagnelie P (1998). Theoretical and applied statistics [Statistiques Théoriques et Appliquées].

[31] Aholoukpè H.S.N, Amadji G.L, Koussihouèdé H.K.I, Chevallier T, Razafimbelo T.M, Chapuis-Lardy L, Brossard M (2020). Carbon stocks in the soils of Benin's agro-ecological zones?. Soil Carbon in Africa:Impacts of Soil Use and Agricultural Practices, Syntheses [Stocks de carbone dans les sols des zones agro-écologiques du Bénin Carbone Des Sols en Afrique:Impacts Des Usages Des Sols et Des Pratiques Agricoles, Synthèses].

[32] R Core Team (2021). R A Language and Environment for Statistical Computing.

[33] Lê S, Josse J, Husson F (2008). FactoMineR:An R Package for Multivariate Analysis. J. Stat. Softw.

[34] Kassambara A, Mundt F (2020). Factoextra:Extract and Visualize the Results of Multivariate Data Analyses. R Package Version 1.0.7 [Factoextra:Extract and Visualize the Results of Multivariate Data Analyses, R Package 1].

[35] Djohy G.L, Boukou B.S, Dossou P.J, Yabi J.A (2021). Perception of climate change by cattle herders and meteorological observations in the Upper Oueme Basin in Benin [Perception des changements climatiques par les éleveurs de bovins et observations météorologiques dans le bassin de l'Ouémésupérieur au Bénin]. Rev. Elev. Med. Vet. Pays Trop. [Rev. D'élevage Méd. Vét. Pays Trop.].

[36] Hessa C.C, Yaya I, Assani A.S, Sanni H.S.W, Alkoiret I.T (2023). Characterization of silvopastoral and agrosilvopastoral cattle farms in Benin [Caractérisation des exploitations d'élevage bovin de types sylvopastoral et agrosylvopastoral au Bénin]. Rev. Marocaine Sci. Agron. Vét.

[37] Onono J, Mutua P, Kitala P, Gathura P (2019). Knowledge of pastoralists on livestock diseases and exposure assessment to brucellosis within rural and peri-urban areas in Kajiado, Kenya. F1000Res.

[38] Ntirandekura J.B, Matemba L.E, Ngowi H.A, Kimera S.I, Karimuribo E.D (2018). Knowledge, perceptions and practices regarding brucellosis in pastoral communities of Kagera Region in Tanzania. J. Adv. Vet. Anim. Res.

[39] Hayatou H, Amarir F.E, Bouslikhane M, Rhalem A, Awah-Ndukum J, Meutchieye F (2023). State of knowledge of ticks and diseases transmitted in beef cattle production production systems in Cameroon, Central Africa [Etat de connaissance des tiques et des maladies transmises dans les systèmes de production de bovins viande au Cameroun, Afrique Centrale]. J. Cameroon Acad. Sci.

[40] Bierschenk T, Forster R (2004). Social organisation of the Peulh in eastern Atacora (Republic of Benin, Commune of Kouandé, Pehonco and Kérou) [L'organisation Sociale des Peulh Dans l'Est de l'Atacora (République du Bénin, Commune de Kouandé, Pehonco et Kérou)]. Arbeitspapiere/Working Papers No 46. Gutemberg University, Mainz.

[41] Aboudou K, Alidou C, Vodouhe-Egueh S, Gougbe-Semako A, Sagbohan G.T, Soumanou M (2021). Technological aptitude of two coagulants (Calotropaine and papain) for the production of Peulh cheese produced in Benin [Aptitude technologique de deux coagulants (Calotropaïne et Papaïne) àla production du fromage peuhl produit au Bénin]. Afr. Sci.

[42] Dahouda M, Boubacar Y, Dossa L.H, Dotche I, Ahounou S, Kiki P, Youssao I (2019). Feeding strategies and management of feed resources on cattle farms in the Communes of Nikki, Kalaléand N'Dali in north-east Benin [Stratégies d'alimentation et gestion des ressources alimentaires dans les élevages bovins des Communes de Nikki, Kalaléet N'Dali au Nord Est Bénin]. Rev. Int. Sci. Appl.

[43] Houndje E.M.B, Ogni C.A, Noudeke N, Farougou S, Youssao A.K.I, Kpodekon T.M (2016). Ethno-veterinary recipes of medicinal plants using for the treatment of foot and mouth disease in Benin [Recettes ethno-vétérinaire àbase de plantes médicinales utilisées pour le traitement de la fièvre aphteuse au Bénin]. Int. J. Biol. Chem. Sci.

[44] Youssao A.K.I, Dahouda M, Attakpa E.Y, Koutinhouin G.B, Ahounou G.S, Toleba S.S, Balogoun B.S (2013). Diversity of Borgou cattle rearing systems in the Sudanian zone of Benin [Diversitédes systèmes d'élevages de bovins de race bovine Borgou dans la zone soudanienne du Bénin]. Int. J. Biol. Chem. Sci.

[45] Chabi Toko R (2016). Cattle farming contribution to Fulani rural economy in northern Benin [Place de L'élevage Bovin Dans L'économie Rurale Des Peuls Du Nord-Bénin]. Dissertation for a PhD in Agricultural Sciences and Biological Engineering [Dissertation Originale Présentée En Vue De L'obtention Du Grade De Docteur En Sciences Agronomiques Et Ingénierie Biologique]. University of Liège - Gembloux Agro-Bio Tech.

[46] Marcotty T, Matthys F, Godfroid J, Rigouts L, Ameni G, Gey van Pittius N, Kazwala R, Muma J, Van Helden P, Walravens K (2009). Zoonotic tuberculosis and brucellosis in Africa:Neglected zoonoses or minor public-health issues?The outcomes of a multi-disciplinary workshop. Ann. Trop. Med. Parasitol.

[47] Kang'ethe E.K, Arimi S.M, Omore A.O, McDermott J.J, Nduhiu J.G, Macharia J.K, Githua A (2007). Testing for antibodies to *Brucella abortus* in milk from consumers and market agents in Kenya using milk ring test and enzyme immunoassay. Kenya Vet.

[48] Kansiime C, Mugisha A, Makumbi F, Mugisha S, Rwego I.B, Sempa J, Kiwanuka S.N, Asiimwe B.B, Rutebemberwa E (2014). Knowledge and perceptions of brucellosis in the pastoral communities adjacent to Lake Mburo National Park, Uganda. BMC Public Health.

[49] Edao B.M, Ameni G, Assefa Z, Berg S, Whatmore A.M, Wood J.L (2020). Brucellosis in ruminants and pastoralists in Borena, Southern Ethiopia. PLoS Negl. Trop. Dis.

[50] Cloete A, Gerstenberg C, Mayet N, Tempia S (2019). Brucellosis knowledge, attitudes and practices of a South African communal cattle keeper group. Onderstepoort J. Vet. Res.

[51] Cadmus S, Salam S.P, Adesokan H.K, Akporube K, Ola-Daniel F, Awosanya E.J (2021). Seroprevalence of brucellosis and Q fever infections amongst pastoralists and their cattle herds in Sokoto State, Nigeria. PLos One.

[52] Madzingira O, Byaruhanga C, Fasina F.O, Van Heerden H (2023). Assessment of knowledge, attitudes and practices relating to brucellosis among cattle farmers, meat handlers and medical professionals in Namibia. Vet. Med. Sci.

[53] Babo S.A, Fokou G, Yapi R.B, Mathew C, Dayoro A.K, Kazwala R.R, Bonfoh B (2022). Gendered asymmetry of access to knowledge for brucellosis control among pastoral communities in north-west Côte d'Ivoire. Pastoralism.

[54] Buhari H.U, Saidu S.N.A, Mohammed G, Raji M.A (2015). Knowledge, attitude and practices of pastoralists on bovine brucellosis in the north senatorial district of Kaduna state, Nigeria. J. Anim. Health Prod.

[55] Nabirye H.M, Erume J, Nasinyama G.W, Kungu J.M, Nakavuma J, Ongeng D, Owiny D.O (2017). Brucellosis:Community, medical and veterinary workers'knowledge, attitudes, and practices in Northern Uganda. Int. J. One Health.

[56] Obonyo M, Gufu W.B (2015). Knowledge, attitude and practices towards brucellosis among pastoral community in Kenya, 2013. Int. J. Innov. Res. Dev.

[57] Musallam I.I, Abo-Shehada M.N, Guitian J (2015). Knowledge, attitudes, and practices associated with brucellosis in livestock owners in Jordan. Am. J. Trop. Med. Hyg.

[58] Díez J.G, Coelho A.C (2013). An evaluation of cattle farmers'knowledge of bovine brucellosis in northeast Portugal. J. Infect. Public Health.

[59] Mufinda F.C, Boinas F.S, Nunes C.S (2015). Prevalence and factors associated with cattle brucellosis in animal herds of the Namibe province in Angola. Alex. J. Vet. Sci.

[60] Njuguna J.N, Gicheru M.M, Kamau L.M, Mbatha P.M (2017). Incidence and knowledge of bovine brucellosis in Kahuro district, Murang'a County, Kenya. Trop. Anim. Health Prod.

[61] Lindahl E, Sattorov N, Boqvist S, Magnusson U (2015). A study of knowledge, attitudes and practices relating to brucellosis among small-scale dairy farmers in an urban and peri-urban area of Tajikistan. PLoS One.

[62] Arif S, Thomson P.C, Hernandez-Jover M, McGill D.M, Warriach H.M, Heller J (2017). Knowledge, attitudes and practices (KAP) relating to brucellosis in smallholder dairy farmers in two provinces in Pakistan. PLoS One.

[63] Kothalawala K.A.C.H.A, Makita K, Kothalawala H, Jiffry A.M, Kubota S, Kono H (2018). Knowledge, attitudes, and practices (KAP) related to brucellosis and factors affecting knowledge sharing on animal diseases:A cross-sectional survey in the dry zone of Sri Lanka. Trop. Anim. Health Prod.

[64] Ruano M.P, Aguayo M.Z (2017). Study of knowledge about bovine brucellosis among people involved in the cattle supply chain in the province of Manabí, Ecuador. Tech.

[65] ANOPER (Association Nationale des Organisations Professionnelles des Eleveurs de Ruminants au Bénin) (2014). The current situation of livestock farming and ruminant breeders in Benin:analysis and outlook, Republic of Benin [La Situation Actuelle De L'élevage Et Des Éleveurs De Ruminants Au Bénin :Analyse et Perspective, République du Bénin].

[66] Kunda J, Fitzpatrick J, Kazwala R, French N.P, Shirima G, MacMillan A, Kambarage D, Bronsvoort M, Cleaveland S (2007). Health-seeking behaviour of human brucellosis cases in rural Tanzania. BMC Public Health.

[67] Zhang N, Zhou H, Huang D.S, Guan P (2019). Brucellosis awareness and knowledge in communities worldwide:A systematic review and meta-analysis of 79 observational studies. PLoS Negl. Trop. Dis.

[68] Sylla S, Sidimé Y, Sun Y, Doumbouya S, Cong Y (2014). Seroprevalence investigation of bovine brucellosis in Macenta and Yomou, Guinea. Trop. Anim. Health Prod.

[69] Alsaif M, Dabelah K, Girim H, Featherstone R, Robinson J.L (2018). Congenital brucellosis:A systematic review of the literature. Vector Borne Zoonotic Dis.

[70] Ducrotoy M.J, Ammary K, Ait Lbacha H, Zouagui Z, Mick V, Prevost L, Bryssinckx W, Welburn S.C, Benkirane A (2015). Narrative overview of animal and human brucellosis in Morocco:Intensification of livestock production as a driver for emergence?. Infect. Dis. Poverty.

[71] Adesokan H.K, Alabi P.I, Stack J.A, Cadmus S.I.B (2013). Knowledge and practices related to bovine brucellosis transmission amongst livestock workers in Yewa, south-western Nigeria. J. S. Afr. Vet. Assoc.

[72] Kiros A, Asgedom H, Abdi R.D (2016). A review on bovine brucellosis:Epidemiology, diagnosis and control options. ARC J. Anim. Vet. Sci.

[73] Tialla D, Koné P, Kadja M.C, Kamga-Waladjo A, Dieng C.B, Ndoye N, Kouame K.G.G, Bakou S, Akakpo A.J (2014). Prevalence of bovine brucellosis and related risk behavior in the suburban area of Dakar, Senegal [Prévalence de la brucellose bovine et comportements àrisque associés àcette zoonose dans la zone périurbaine de Dakar au Sénégal]. Rev. Elev. Med. Vet. Pays Trop. [Rev. D'élevage Méd. Vét. Pays Trop.].

[74] Aparicio E.D (2013). Epidemiology of brucellosis in domestic animals caused by *Brucella melitensis*, *Brucella suis* and *Brucella abortus*. Rev. Sci. Tech.

[75] Ducrotoy M, Bertu W.J, Matope G, Cadmus S, Conde-Álvarez R, Gusi A.M, Welburn S, Ocholi R, Blasco J.M, Moriyón I (2017). Brucellosis in Sub-Saharan Africa:Current challenges for management, diagnosis and control. Acta Trop.

[76] Adesokan H.K, Alabi P.I, Ogundipe M.A (2016). Prevalence and predictors of risk factors for Brucellosis transmission by meat handlers and traditional healers'risk practices in Ibadan, Nigeria. J. Prev. Med. Hyg.

[77] Mangesho P.E, Caudell M.A, Mwakapeje E.R, Ole-Neselle M, Kabali E, Obonyo M, Dorado-Garcia A, Valcarce A, Kimani T, Price C (2021). “We are doctors”:Drivers of animal health practices among Maasai pastoralists and implications for antimicrobial use and antimicrobial resistance. Prev. Vet. Med.

